# Effector-Mining in the Poplar Rust Fungus *Melampsora larici-populina* Secretome

**DOI:** 10.3389/fpls.2015.01051

**Published:** 2015-12-15

**Authors:** Cécile Lorrain, Arnaud Hecker, Sébastien Duplessis

**Affiliations:** ^1^INRA, UMR 1136 Interactions Arbres/Microorganismes INRA/Université de Lorraine, Centre INRA Nancy Lorraine, Champenoux, France; ^2^Université de Lorraine, UMR 1136 Interactions Arbres/Microorganismes Université de Lorraine/INRA, Faculté des Sciences et Technologies, Vandoeuvre-lès-Nancy, France

**Keywords:** effector protein, poplar rust, prediction pipeline, expert annotation, multigene families analysis

## Abstract

The poplar leaf rust fungus, *Melampsora larici-populina* has been established as a tree-microbe interaction model. Understanding the molecular mechanisms controlling infection by pathogens appears essential for durable management of tree plantations. In biotrophic plant-parasites, effectors are known to condition host cell colonization. Thus, investigation of candidate secreted effector proteins (CSEPs) is a major goal in the poplar–poplar rust interaction. Unlike oomycetes, fungal effectors do not share conserved motifs and candidate prediction relies on a set of *a priori* criteria established from reported *bona fide* effectors. Secretome prediction, genome-wide analysis of gene families and transcriptomics of *M. larici-populina* have led to catalogs of more than a thousand secreted proteins. Automatized effector-mining pipelines hold great promise for rapid and systematic identification and prioritization of CSEPs for functional characterization. In this review, we report on and discuss the current status of the poplar rust fungus secretome and prediction of candidate effectors from this species.

## Introduction

Filamentous plant pathogens use secreted molecules for manipulating immunity and physiology of their hosts (Jones and Dangl, 2006; Kamoun, 2009; Win et al., 2012; Okmen and Doehlemann, 2014). Among these, secreted proteins and secondary metabolites can be defined as key players in the outcome and stability of host-parasite interactions with very diverse functions (MacLean et al., 2014; Rovenich et al., 2014; Lo Presti et al., 2015; Pusztahelyi et al., 2015). Chemical effectors (i.e., secondary metabolites) are secreted mainly by necrotrophs and hemibiotrophs during their necrotrophic phase (Kemen et al., 2015). Obligate biotrophs are organisms that grow, feed and reproduce on living host tissues. They exhibit small or very reduced sets of genes encoding secondary metabolites and cell-wall degrading enzymes while they possess large repertoires of effector proteins (Duplessis et al., 2014a; Kemen et al., 2015). In the case of obligate biotrophs such as rust fungi, investigations have largely focused on secreted proteins (SPs) of plant-associated organisms (i.e., the secretome) with potential for being candidate secreted effector proteins (CSEPs).

Rust fungi (Pucciniales, Basidiomycetes) are among the most studied fungal obligate biotrophs due to the degree to which they cause damage to many cultivated plants (Dean et al., 2012). Rust fungi are physically associated with their host cells through the formation of specialized infection structures called haustoria, which are known as secretion sites for effector proteins (Rafiqi et al., 2012; Petre and Kamoun, 2014). The biotrophic life style of rust fungi prohibits virtually all growth on synthetic media and makes genetic transformation very difficult to achieve. Therefore very little is known about the molecular mechanisms underlying the colonization of host tissues by rust fungi and to date, only six rust fungi effector proteins have been reported (see Petre et al., 2014 for review). Next generation sequencing technologies have provided access to genomes or transcriptomes for several rust fungi (Duplessis et al., 2014b). So far, genomes of five rust fungi have been published: the poplar rust *Melampsora larici-populina*, the wheat stem rust *Puccinia graminis* f. sp. *tritici*, the wheat stripe rust *Puccinia striiformis* f. sp. *tritici*, the flax rust *Melampsora lini* and the coffee rust *Hemileia vastatrix* (Cantu et al., 2011, 2013; Duplessis et al., 2011a; Zheng et al., 2013; Cristancho et al., 2014; Nemri et al., 2014). Secretomes of rust fungi have been determined based on the presence of predicted N-terminal signal peptides in proteins (Cantu et al., 2011, 2013; Duplessis et al., 2011a; Fernandez et al., 2012; Hacquard et al., 2012; Saunders et al., 2012; Bruce et al., 2013; Garnica et al., 2013; Zheng et al., 2013; Link et al., 2014; Nemri et al., 2014). Signal peptides can be defined using predictors available online (Emanuelsson et al., 2007). These predictions have revealed the presence of a plethora of SPs in rust fungal species. A recent comparison of genomic features in 84 plant-associated fungi has shown that the proteomes of obligate biotrophs are enriched in SPs, most of which are of unknown function (Lo Presti et al., 2015). This illustrates the importance of studying rust secretomes for identifying potential CSEPs.

**FIGURE 1:**
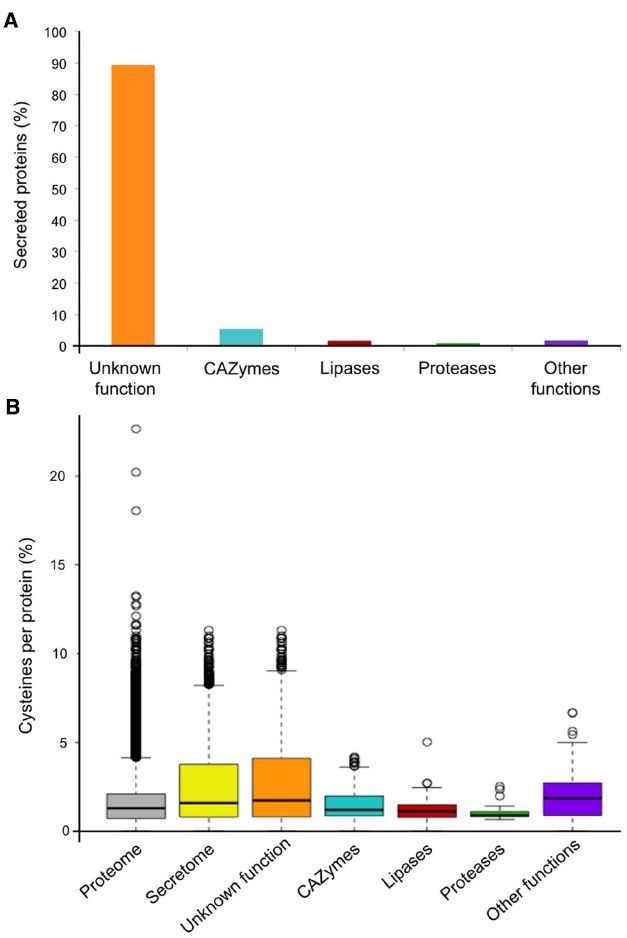
*****M. larici-populina*** predicted secretome and cysteine content. (A)** Composition of the *M. larici-populina* predicted secretome. Bars indicate the percentage of predicted secreted proteins (SPs): proteins of unknown function (orange), carbohydrate active enzymes (CAZymes; blue), lipases (red), proteases (green) and other functions (purple). The secretome prediction reported here has been updated based on the current version of the poplar rust genome available at the Joint Genome Institute Mycocosm (May 2015; http://genome.jgi-psf.org/programs/fungi). Annotation of SP functions was based on the expert curation reported in Duplessis et al. (2011a) and updated for CAZymes, Lipases and Proteases. **(B)** Cysteine content in *M. larici-populina* SPs. The percentage of cysteine residues per proteins are symbolized by box plots where the top and bottom of the boxes show the 25 and 75% quartiles, respectively, and the middle line indicates the median (50% quartile). The bottom whisker corresponds to the 1.5 interquartile range of the lower quartile and the top whisker indicates the 1.5 interquartile range of the upper quartile. White circles represent outliers.

The poplar leaf rust fungus *M. larici-populina* causes annual epidemics and severe damage to Northern European poplar plantations. Investigations of the poplar–poplar rust pathosystem using “-omic” approaches have led to significant progress in describing this interaction (Hacquard et al., 2011). The genome of *M. larici-populina* was one of the first rust genomes to be sequenced by an international research consortium (Duplessis et al., 2011a). *In sillico* genome annotation and secretome prediction have been instrumental in unraveling *M. larici-populina* SPs. Among the 16,399 predicted protein-coding genes reported in the poplar rust genome, 13.3% are predicted SPs (2168 SPs) of which 89.3% have unknown functions (Figure 1A). Other secreted proteins correspond to carbohydrate active enzymes (5.8%), lipases (2.3%), proteases (0.8%), and other functions (1.8%) (Figure 1A). Extensive genomic and transcriptomic studies have also positioned *M. larici-populina* as being a model tree pathogen for molecular investigations (Hacquard et al., 2010, 2012, 2013; Joly et al., 2010; Duplessis et al., 2011a,b; Petre et al., 2012; Pernaci et al., 2014; Persoons et al., 2014).

### From *M. larici-populina* SPs to CSEPs: Post-genomic Strategies

**FIGURE 2:**
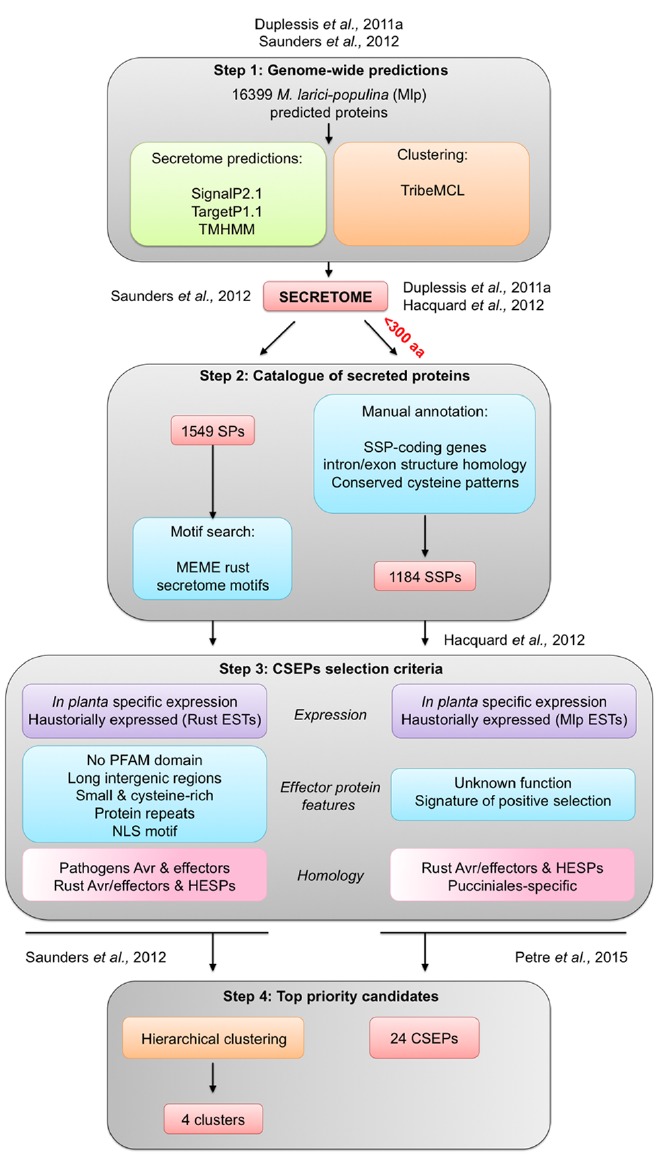
**Overview of effector-mining pipelines applied to ***M. larici-populina*** secretome to prioritize candidate secreted effector proteins (CSEPs).** The pipelines can be divided in four main steps: step 1 *Genome-wide predictions* identifies the *M. larici-populina* secretome using prediction tools (green) and gene families clustering with TribeMCL (orange); step 2 *Catalog of secreted proteins* establishes a set of secreted proteins; step 3 *CSEPs selection criteria* identifies CSEPs using different features and criteria; and step 4 *Top priority candidates* prioritizes CSEPs for further functional characterization. See text for details. Avr, avirulence protein; EST, expressed sequence tag; SP, secreted protein; SSP, small secreted protein; HESP, haustorially expressed secreted protein.

Two independent studies have defined different pipelines to pinpoint priority poplar rust CSEPs from catalogs of predicted SPs (Figure 2; Hacquard et al., 2012; Saunders et al., 2012). In both studies, *M. larici-populina* secretome was predicted using the same prediction tools. SignalP2.1 was used to sort SPs from the proteome, TargetP1.1 to identify proteins likely retained inside fungal cells (e.g., in mitochondria; Emanuelsson et al., 2000) and TMHMM to exclude proteins carrying transmembrane α-helix domains (Moller et al., 2001; Figure 2). Considering that rust fungal genomes exhibit expanded lineage-specific multigene families compared to other Basidiomycetes, one study used the similarity-based Markov clustering TribeMCL program to group SPs in tribes to further investigate multigene families in *M. larici-populina* and *P. graminis* f. sp. *tritici* (Enright et al., 2002; Saunders et al., 2012). The second study also utilized TribeMCL clustering but added a second level of annotation with expert curation of *M. larici-populina* SP genes. This led to the definition of SP gene families (Duplessis et al., 2011a; Hacquard et al., 2012). It is worth noting that these studies used different parameters and fungal species to perform the TribeMCL clustering. By doing so, the initial repertoire of *M. larici-populina* SPs was shown to differ between the two studies (Figure 2).

To effectively treat the wide range of predicted SPs in the poplar rust fungus and considering the high divergence and absence of conserved motifs in rust *bona fide* effectors, both effector-mining pipelines focused on *a priori* features of plant pathogen effectors (Hacquard et al., 2012; Saunders et al., 2012). Criteria such as expression during infection or in purified haustoria were applied to prioritize candidates. Moreover, the authors utilized other features such as the size of proteins, the content in cysteine residues, the presence of selection signatures (i.e., genes evolving under the pressure of host resistances), homology to known rust fungi effectors and/or previously reported haustorially expressed secreted proteins (HESPs), as well as organization in genes families taking into account specificity at a given taxonomical level (species, genera, family, order; for review, see Petre et al., 2014).

Effector proteins are often described as small proteins (Stergiopoulos and de Wit, 2009; Tyler and Rouxel, 2012). Based on this observation, an arbitrary cut-off can be applied in CSEPs-mining studies to only focus on small secreted proteins (SSPs), although large effector proteins have also been reported (Petre et al., 2014; Lo Presti et al., 2015). The *Melampsora Genome Consortium* performed a manual curation of *M. larici-populina* SSP gene families (i.e., <300 amino acids) taking advantage of expressed sequence tags (ESTs) from haustoria and rust-infected poplar leaves (Joly et al., 2010; Duplessis et al., 2011a; Figure 2). Dedicated expert annotation led to the elimination and the addition of several genes encoding SSPs, and notably generated 170 SSPs that had not previously been predicted by automatic annotation (Duplessis et al., 2011a). Manually annotated SSPs were specifically enriched in cysteine residues (on average 2.8% cysteines per protein compared to 1.6% in the whole proteome; Figure 1B). SSPs with unknown functions were also clearly enriched in cysteine residues compared with annotated SPs (Figure 1B). The proportion of cysteine residues in effectors can indicate the presence of intra-molecular disulfide bridges that could contribute to stabilizing protein structure in inhospitable apoplastic environments (Stergiopoulos et al., 2013). For instance, the *Cladosporium fulvum* Avr2 fungal effector is a cysteine-rich protein, playing a key role in apoplastic protease inhibition during the interaction with tomato leaf cells (Rooney et al., 2005). Although, we cannot definitively dismiss the possibility that cysteine residues could potentially play an important role in the structure of the proteins at their final destination in the host cell. Detailed analysis of SSP gene families taking into account intron/exon organization and cysteine codon positions have revealed certain conserved cysteine patterns (Hacquard et al., 2012). For instance, the largest SSP family composed of 111 members shares the conserved YxC//CxxY//YxC cysteine pattern (Duplessis et al., 2011a; Hacquard et al., 2012). This pattern is reminiscent of the Y/F/WxC motif reported in powdery mildew and wheat rust fungi (Godfrey et al., 2010). Interestingly, other obligate biotrophs also exhibit large repertoires of SPs, such as the white rust oomycete *Albugo laibachii* in which CHXC and CXHC cysteine-rich motifs are found (Kemen et al., 2011). Both types of patterns were speculated to be potentially involved in delivery of effector into host cells.

In the study conducted by Hacquard et al. (2012), numerous effectors features were considered to facilitate selection of the most promising CSEPs: specific expression in infected host tissues, unknown function, homology to known rust effectors and HESPs, specificity to the Pucciniales order and signatures of positive selection (Figure 2). Effector-mining studies often use evidence expression during host interaction as a filter to identify critical CSEPs (Lo Presti et al., 2015). Time-course of poplar leaf infection by *M. larici-populina* has revealed dynamic patterns of SSP expression during the early stages of infection, biotrophic growth and sporulation (Duplessis et al., 2011b; Hacquard et al., 2012). Expression in resting and germinating spores can be used to differentiate SSP genes specifically expressed *in planta*. The extensive knowledge of *M. larici-populina* SSP expression at different stages of the life cycle is also critical to pinpoint CSEPs (for detailed reviews, see Duplessis et al., 2012 and Duplessis et al., 2014b). The study performed by Hacquard et al. (2012) did not result in a defined list of CSEPs that might be prioritized for future investigation, but it did provide a comprehensive depiction of the complete repertoire of *M. larici-populina* SSP genes.

Manual curation of large fungal genomes such as rust fungi remains a time-consuming process and automatized pipelines could help to foster CSEP detection. The pipeline built by Saunders and collaborators was initially designed to scrutinize the secretomes of *M. larici-populina* and *P. graminis* f. sp. *tritici* (Saunders et al., 2012). It has subsequently been applied to mine the genomes of different fungi interacting with plants, including the wheat rust *P. striiformis* f. sp. *tritici* and the flax rust *M. lini* (Cantu et al., 2013; Lin et al., 2014; Nemri et al., 2014). This *in sillico* pipeline computes Markov clustering generated tribes taken from available genome annotations (Saunders et al., 2012). This pipeline considers three levels of information for SPs: functional annotation, detection of novel effector motifs and annotation of effector features (Figure 2). Most effectors do not have PFAM domains (Kamoun, 2007; Dodds et al., 2009; Stergiopoulos and de Wit, 2009). The functional annotation step allows the selection of SPs with no conserved protein domain families (PFAM), with the exception of avirulence proteins that may have such domains. For instance, the Chitin Binding Module like of Avr4 and the LysM domain of Ecp6 in *C. fulvum* both have PFAM annotations. In total, five PFAM domains were found in rust fungi and were considered for their obvious connection with pathogenicity (Saunders et al., 2012). In a second step, the MEME tool was applied to detect *de novo* conserved patterns in rust SPs (Bailey et al., 2009). Among identified motifs, five motifs containing one or two conserved cysteine residues with high positional constraints in SP tribes were highlighted (Figure 2 Saunders et al., 2012). Interestingly, some motifs such as the motif 06 YxCxYxxCxW, were also identified by the manual annotation of *M. larici-populina* SSP families (Duplessis et al., 2011a; Hacquard et al., 2012). In a final step, common effector features were examined in details, which included induction of expression during host infection, gaging similarity to haustorial ESTs, and determining small protein size (<150 amino acids), content in cysteine residues and known effector motifs or repeats in protein. It has been reported that some effectors contain nuclear localization signals (NLS), suggestive of a potential nuclear localization in host cells (Kanneganti et al., 2007; Shen et al., 2007; Schornack et al., 2010; Liu et al., 2011). The presence of such NLS was also added to the features tested. It was shown for different filamentous plant pathogens such as the fungus *Leptosphaeria maculans* and the oomycete *Phytophthora infestans* that effector genes reside in gene-scarce regions marked by the presence of repeat elements such as transposable elements (Haas et al., 2009; Rouxel et al., 2011; for a review see Raffaele and Kamoun, 2012). This criterion was also taken into account in the pipeline by looking at the presence of long intergenic regions around SP genes. However, no significant link could be established between SSP genes and repeat-rich regions in the genomes *M. larici-populina* and *P. graminis* f. sp. *tritici* (Duplessis et al., 2011a). Nonetheless, all these filters are informative and can be computed in a complex matrix comparing rust tribes in order to rank CSEP (Figure 2; Saunders et al., 2012).

### A Priori Criteria to Prioritized CSEPs

While it may be possible to infer typical features from effector proteins, a given effector will rarely exhibit a combination of all features at the same time. Conversely, each feature will display a diverse distribution among SP families. Thereby, hierarchical clustering can be performed for ranking tribes with the highest probability of containing CSEPs (Saunders et al., 2012). When using this type of clustering approach, the weight of each criterion requires adjustment. By doing this, Saunders and collaborators were able to derive four clusters with the most promising SP tribes that could potentially correspond to CSEPs for further investigation. The largest tribe, with 92 members in one of these clusters, is specific to *M. larici-populina* and contains a large proportion of secreted proteins (73% with predicted signal peptide; Saunders et al., 2012). This tribe corresponds to the largest poplar rust SSP family (with 111 members) as reported by Duplessis et al. (2011a). This SSP family is marked by the presence of highly conserved cysteine patterns, which both studies highlighted. The difference in numbers of SSP members likely corresponds to the different levels of gene annotation considered for the *M. larici-populina* genome. The two studies identified tribes composed of SPs/SSPs and proteins without predicted signal peptides. In some tribes, SPs exhibited homology to HESPs, as well as known rust effector proteins (e.g., *M. lini* ArvM). It could be speculated that such proteins are involved in haustoria functions (Saunders et al., 2012). It could also reflect the evolution of these families with a gain or a loss of signal peptide toward the generation of new putative effector functions.

In a recent study, an effectoromic pipeline identified priority *M. larici-populina* CSEPs for expression in *Nicotiana benthamiana* as a heterologous system to study their localization in plant subcellular compartments and to identify potential plant interactors (Petre et al., 2015a). The pipeline was applied to the catalog of SSPs previously reported by Hacquard et al. (2012). Priority CSEPs were selected by giving a stronger weight to some of the typical criteria used in the two studies reported above. For instance, expression in haustoria, specific induction of expression during poplar infection, specificity to the Pucciniales and proteins of unknown functions were the most important features considered. These criteria were similar to those systematically applied for tribe ranking in Saunders et al. (2012). Redundant family members were also removed in order to focus on orphan and lineage-specific CSEPs, considering that pathogenicity mechanisms imply highly specific functions. Such stringent criteria led to a subset of 24 priority CSEPs from 1184 initial *M. larici-populina* SSPs (Petre et al., 2015a). Among these, only three belong to priority clusters identified in the study by Saunders et al. (2012), in which an initial postulate was to focus on tribes and not on orphan genes. One supposed orphan SSP (CSEP 107772) finally proved to be member of a *M. larici-populina* multigene family (Petre et al., 2015b). This particular example illustrates how automatic and dedicated pipelines strongly depend on the accuracy of genome annotation and parameters applied to gene family analysis tools, above any further selection criteria. Among the 24 selected CSEPs, 20 could be expressed in fusion with GFP in *N. benthamiana*, which further allows identification of specific localisation in plant cell compartments as well as potential plant interactors through coimmunoprecipitation and mass spectrometry. This study identified six *M. larici-populina* CSEPs with a specific localization pattern (i.e., nucleus, nucleolus, chloroplast, mitochondria, and cytosolic bodies) and five were specifically associated with plant proteins representing potential interactors (Petre et al., 2015a). The development of *in planta* assays in heterologous systems has provided the first step toward effector characterization for various pathogens (Bozkurt et al., 2011; Caillaud et al., 2012, 2013; Petre et al., 2015a,b). Another alternative already proven successful for different obligate biotrophic fungi, including rust fungi, is host-induced gene silencing (Panwar et al., 2013; Spanu, 2015). Knowledge of effector structure is useful to understand effector/plant protein interactions and to find structure homology within effectors (Maqbool et al., 2015). *In vitro* structural biology of effectors is also an option of choice to further determine the role of effectors in rust fungi, pending fruitful production of recombinant CSEPs.

Pucciniales genomes are marked by the presence of very large catalogs of SPs. Among these only a fraction may be *bona fide* effectors. Effector-mining pipelines, while still imperfect, are crucial to highlighting the most crucial CSEPs in these fungal species. No robust ubiquitous effector motifs have been found in rust fungal effectors, contrary to the RXLR motif of oomycetes effectors (Petre and Kamoun, 2014; Sperschneider et al., 2015). As illustrated here, effector mining in the poplar rust fungus relies both on the quality of input data (i.e., gene annotation and gene families analysis) and on several qualitative and subjective criteria. Indeed, there is no absolute rule for determining rank and weight among the many effector features that may be considered. As illustrated here, knowledge about SP gene expression is a key criterion for selecting priority CSEPs. Considering the complex life cycle of heteroecious rust fungi like *M. larici-populina* (i.e., alternation on two different hosts and production of five different spores), insight of gene expression patterns throughout the entire life cycle can help to drastically reduce the overall catalog of CSEPs. Pucciniales consist of more than 8000 species (Aime et al., 2014). To date, less than 10 genomes have been published or sequenced with partial data available (see Duplessis et al., 2014b for review). All rust fungal genomics and transcriptomics reports have shown that these species contain a high content of Pucciniales or species-specific genes. There is a strong probability that rust fungi possess highly specific effectors and increasing the amount of genomic data will surely help to focus efforts toward CSEPs identification. Still highly anticipated is future development of dedicated bioinformatics tools for predicting fungal effectors (Sperschneider et al., 2015).

## Author Contributions

All the authors wrote and revised the manuscript.

### Conflict of Interest Statement

The authors declare that the research was conducted in the absence of any commercial or financial relationships that could be construed as a potential conflict of interest.
